# Schlafen 5 is an intracellular immune checkpoint and controls IFN responses in pancreatic ductal adenocarcinoma

**DOI:** 10.1172/jci.insight.190031

**Published:** 2026-01-27

**Authors:** Mariafausta Fischietti, Markella Zannikou, Elspeth M. Beauchamp, Diana Saleiro, Aneta H. Baran, Briana N. Hryhorysak, Jamie N. Guillen Magaña, Emely Lopez Fajardo, Gavin T. Blyth, Brandyn A. Castro, Jason M. Miska, Catalina Lee-Chang, Priyam Patel, Elizabeth T. Bartom, Masha Kocherginsky, Frank Eckerdt, Leonidas C. Platanias

**Affiliations:** 1 Robert H. Lurie Comprehensive Cancer Center of Northwestern University, Chicago, Illinois, USA.; 2 Division of Hematology/Oncology, Department of Medicine, Feinberg School of Medicine, Northwestern University, Chicago, Illinois, USA.; 3 Department of Medicine, Jesse Brown Veterans Affairs Medical Center, Chicago, Illinois, USA.; 4 Department of Neurological Surgery,; 5 Department of Biochemistry and Molecular Genetics, and; 6 Division of Biostatistics, Department of Preventive Medicine, Feinberg School of Medicine, Northwestern University, Chicago, Illinois, USA.

**Keywords:** Cell biology, Oncology, Cancer, Cytokines

## Abstract

We provide evidence that human and murine Schlafen 5 (SLFN5) proteins are modulators of type I IFN responses and the immune response in pancreatic ductal adenocarcinoma (PDAC). Blocking expression of *Slfn5* in PDAC enhanced IFN responses, suppressed tumor growth, and prolonged survival in immunocompetent mice. Notably, immunophenotypic analysis revealed a reduction in tumor-associated macrophages alongside an increase in tumor-infiltrating effector cells in tumors over time. These findings suggest SLFN5 acts as an intracellular immune checkpoint and identify it as a unique therapeutic target for the development of therapies for PDAC and possibly other malignancies.

## Abstract 2

*Slfn5* is target and modulator of Type I IFN responses, and promotes an immunosuppressive tumor microenvironment.

## Introduction

Pancreatic ductal adenocarcinoma (PDAC) is a devastating gastrointestinal epithelial cancer with exceedingly poor prognosis and minimal responsiveness to current immune therapies (1, 2). Despite recent advancements in immunomodulatory treatments for other cancers, PDAC has remained refractory to such interventions, largely due to its tumor microenvironment (TME) that is defined by increased infiltration of immunosuppressive cells and reduced cytotoxic effector cells promoting immune evasion (2). The refractoriness and poor prognosis of PDAC underscores the need for the development of unique new approaches to overcome immunotherapy resistance in PDAC.

In previous work, we demonstrated that expression of a member of the Schlafen (SLFN) family of proteins, SLFN5, correlates with poor prognosis in PDAC, while its disruption inhibits PDAC tumor growth (3). The SLFN family of genes and proteins was initially identified in mice, based on their cell cycle suppressive effects on thymocytes (4). Since their original description, extensive studies have provided evidence for roles of SLFNs in disease-relevant processes such as antiviral responses (5–9), regulation of tumor growth and survival (3, 10–16), sensitivity to chemotherapy (17–24), cell cycle progression (25–27), and stemness and differentiation (14, 28–31). SLFNs represent a family of tandemly arrayed paralogs, with mouse *Slfn* genes *Slfn1*, -*2*, -*3*, -*4*, -*5*, -*8*, -*9*, and -*14* and pseudogene *Slfn10* clustering on chromosome 11 (32, 33), and human *SLFN* genes *SLFN5*, -*11*, -*12*, -*12L*, -*13*, and -*14* on chromosome 17 (32, 33). Although sequence alignment analysis suggested that murine SLFN5 and SLFN14 proteins may represent direct orthologs of human SLFN5 and SLFN14, a conserved orthological function in complex biological systems has not yet been determined (32, 34).

Mechanistically, SLFN5 promotes S-phase progression in PDAC through interaction with the cell cycle regulator E2F7 (3), and this cell cycle–promoting role of SLFN5 has been corroborated by others (26). Remarkably, we demonstrated that genetic disruption of human *SLFN5* elicited potent antitumor responses in immunocompromised preclinical xenograft mouse models of glioblastoma (GBM) and PDAC (3, 13), both of which are considered immunologically “cold” tumors (35) and resistant to immune checkpoint blockade (ICB) (36, 37).

*SLFN5* is an IFN-stimulated gene in malignant and immune cells (3, 13, 15, 38). IFNs exert essential roles in anticancer immune responses (39–43). Importantly, IFNs trigger concurrent antitumor and immunomodulatory effects (reviewed in ref. 44). To avoid continuous IFN effects and limit excessive IFN-induced inflammatory responses, IFNs induce a network of negative IFN regulators to limit inflammatory signaling (45, 46). SLFN5 is both target and suppressor of IFN signaling and we previously suggested that it may be functioning as an “intracellular immune checkpoint” due to its ability to curb transcription of IFN-stimulated genes (ISGs), thereby blunting IFN responses (46). Thus, we have hypothesized that targeting SLFN5 could simultaneously block malignant cell proliferation and enhance IFN-mediated antitumor immune responses in immunologically cold tumors. PDAC is one such immunologically cold tumor with rising incidence and high mortality, expected to emerge as the second most common cause of cancer-related deaths in the United States over the next decade (47–49). Because of the extremely poor prognosis and limited therapeutic options (50), SLFN5 may represent a promising target for immune-based strategies to overcome PDAC’s microenvironment-driven resistance.

## Results

### Loss of SLFN5 modulates IFN-α–induced target gene transcription in human PDAC cells.

**Figure 1:**
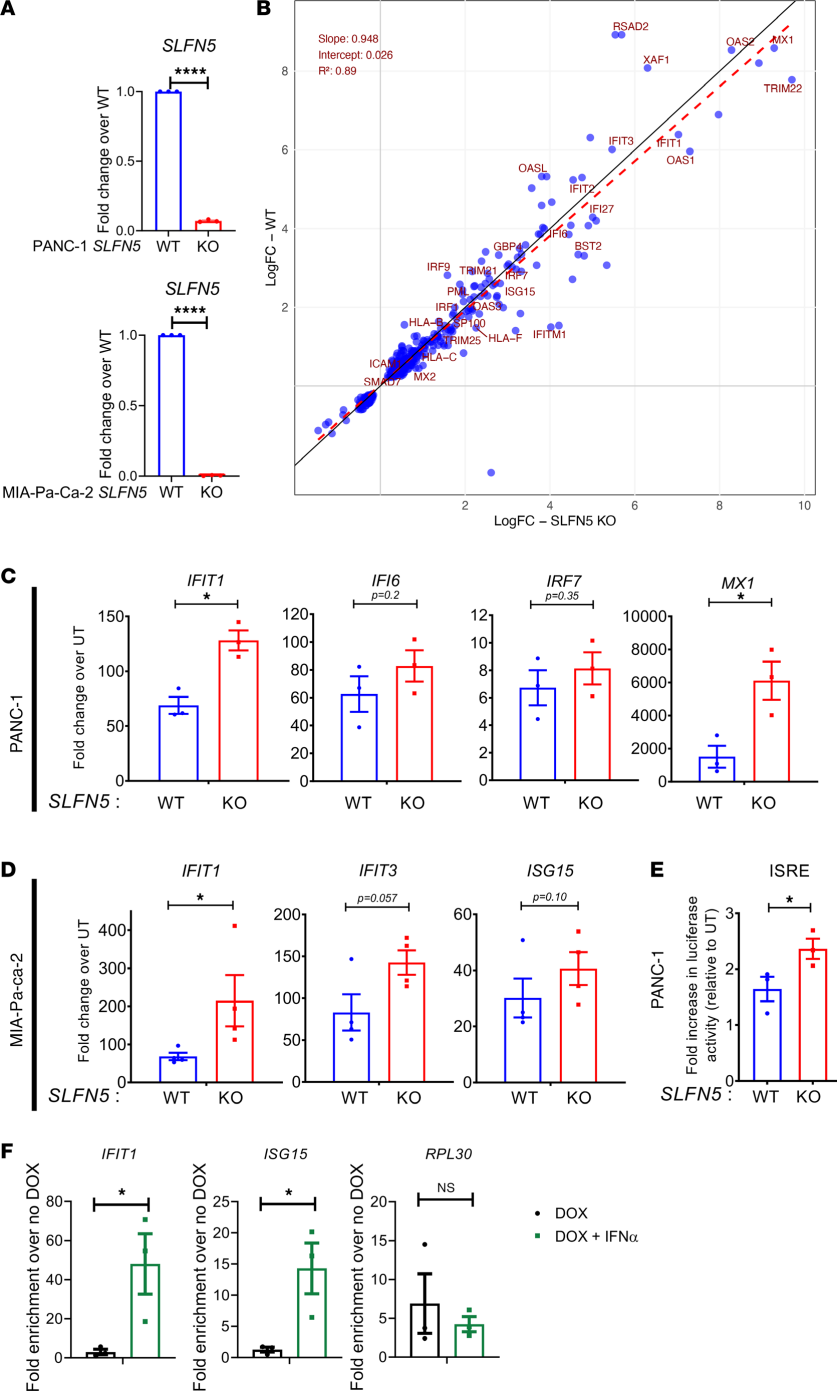
Genetic deletion of human *SLFN5* enhances type I IFN transcriptional responses in PDAC cells. (**A**) RT-qPCR analysis to monitor efficacy of CRISPR/Cas9-mediated *SLFN5* KO in PANC-1 (upper panel) and MIA-Pa-Ca-2 (lower panel) cells. Data are expressed as fold change over WT controls, and bar graphs represent mean ± SEM of 3 independent experiments. ****P < 0.0001. (**B**) Scatter plot (derived from high-throughput single-end RNA-seq analysis) showing the relationship between log_2_(fold change) (LFC) of 347 genes differentially expressed in both *SLFN5*-KO (*x* axis) and WT (*y* axis) PANC-1 cells following IFN-α (5,000 IU for 6 hours) treatment. Select IFN-stimulated genes (ISGs) from the Reactome database (https://www.reactome.org/content/detail/R-HSA-913531; identifier R-HAS-913531) are indicated. The red dashed line deviates from the unity line (*y* = *x*, black line) and represents the linear regression fit (*y* = 0.948*x* + 0.026), capturing the overall trend between the 2 conditions. The slope (*m* = 0.948) indicates a near one-to-one correspondence between the conditions, and the intercept (*c* = 0.026) suggests minimal baseline difference. The high coefficient of determination (*R*^2^ = 0.89) reflects that 89% of the variance in WT LFCs is explained by *SLFN5*-KO LFCs. (**C** and **D**) RT-qPCR analyses of relative mRNA expression of the indicated ISGs in *SLFN5*-WT and -KO PANC-1 (**C**) and MIA-Pa-Ca-2 (**D**) cells untreated or treated with human IFN-α (5,000 IU, 6 hours). *GAPDH* was used for normalization and as an internal control. The data are expressed as fold change over the corresponding untreated cells; bar graphs represent mean ± SEM of 3 (**C**) or 4 (**D**) independent experiments. *P < 0.05. (**E**) PANC-1 *SLFN5*-WT and-KO cells were stably transduced with an ISRE-luciferase promoter construct. Cells were incubated for 6 hours in the presence or absence of human IFN-α (5,000 IU) and luciferase activity was measured. Data are expressed as fold increase in luciferase activity in response to IFN-α treatment over control untreated samples for each condition. Bar graphs show mean ± SEM of 3 independent experiments. *P = 0.05. (**F**) ChIP for SLFN5 in PANC-1 cells transduced with lentivirus carrying doxycycline-inducible SLFN5-MYC-FLAG fusion construct. Cells were grown in the presence or absence of doxycycline for 48 hours, followed by IFN-α treatment for 6 hours. qPCR was performed on immunoprecipitated DNA with primers for the ISRE elements in the *IFIT1* or *ISG15* promoter. Primers for the *RPL30* promoter were used as control. Data were normalized to their own IgG control and are expressed as fold enrichment over doxycycline-untreated cells. Shown are mean ± SEM of 3 independent experiments. *P < 0.05. Significance assessed by 2-tailed unpaired *t* test with Welch’s correction (**A**) or 1-tailed unpaired *t* test with Mann-Whitney test (**C**–**F**).

In order to investigate the role of SLFN5 in type I IFN transcriptional responses in the context of pancreatic cancer, we employed established *SLFN5*-knockout (*SLFN5*-KO) PDAC cell lines (PANC-1 and MIA-Pa-Ca-2) that we previously generated (3). After we confirmed again the relevant *SLFN5* KO (Figure 1A), in initial experiments, we subjected total RNA isolated from untreated and IFN-α–treated *SLFN5*-KO PANC-1 cells and corresponding WT counterparts to high-throughput single-end RNA-sequencing (RNA-seq) analysis. Principal component analysis (PCA) indicated the biological replicates within each of the 4 experimental groups clustered together with a low variance and all 4 groups clustered separately as distinct experimental groups (Supplemental Figure 1A; supplemental material available online with this article; https://doi.org/10.1172/jci.insight.190031DS1). Further comparative transcriptomic analysis revealed 1345 genes that were differentially expressed at significance (FDR-adjusted *P* < 0.05) after IFN-α treatment in *SLFN5*-KO cells, and only 563 genes in WT cells (Supplemental Table 1), with 347 differentially regulated genes in both *SLFN5*-KO and WT PANC-1 cells (Supplemental Table 1). When these 347 genes were plotted for log_2_(fold change) (LFC), the linear regression analysis revealed a slope (*m*) of 0.948, indicating that changes in *SLFN5* KO are slightly more pronounced than in WT (Figure 1B). This tilt of the regression line (red dashed line) toward the *SLFN5*-KO axis becomes apparent when compared to the unity line (*y* = *x*, black line), highlighting a stronger response in *SLFN5* KO relative to WT. Also, analysis of the transcriptomic profiles revealed 199 genes that exhibited at least 2-fold increased expression in *SLFN5*-KO cells after IFN-α treatment (Supplemental Table 2), and ontology analysis indicated that these 199 genes were mostly part of groups related to the response to viruses and IFN signaling (Supplemental Figure 1B). Together, these findings suggest a repressor role for *SLFN5* in IFN responses in PDAC that warrants further investigation. Indeed, this trend toward increased expression of selected ISGs in *SLFN5*-KO cells was confirmed by real-time quantitative PCR (RT-qPCR) for PANC-1 (Figure 1C) and MIA-Pa-Ca-2 (Figure 1D) cells. These results suggest that SLFN5 may act in a repressive manner on certain ISGs’ regulatory elements that are under the control of type I IFN signaling.

### SLFN5 associates with and represses activity of ISRE-containing promoters.

To define the mechanisms by which SLFN5 exerts its repressor effects on ISGs, we next performed luciferase reporter gene assays using PANC-1 cells stably expressing IFN-stimulated response element (ISRE)–Luciferase-pGF1. We found that loss of SLFN5 further enhanced IFN-α–mediated transcriptional responses that are under the control of ISRE (Figure 1E). These results raised the possibility that, in PDAC cells, SLFN5 may exert a repressor role on ISRE promoter elements. Hence, we employed our recently established doxycycline-inducible FLAG-tagged *SLFN5*-overexpressing PANC-1 cell line (3) to assess SLFN5 binding to ISRE promoters in chromatin immunoprecipitation (ChIP) experiments. For this approach, we designed primers for the ISRE sites in the *IFIT1* and *ISG15* promoters. FLAG-ChIP experiments revealed significantly increased enrichment of FLAG-SLFN5 at the promoters of *IFIT1* and *ISG15* (Figure 1F, left and middle panel). We used primers for the *RPL30* promoter as control and did not detect IFN-α–induced enrichment of FLAG-SLFN5 at this promoter (Figure 1F, right panel). Together, these findings provide strong evidence for a suppressive role of SLFN5 on type I IFN–stimulated gene expression that involves ISRE repressor activity in pancreatic cancer cells.

### Murine SLFN5 acts as a functional ortholog of human SLFN5 in PDAC cells.

**Figure 2:**
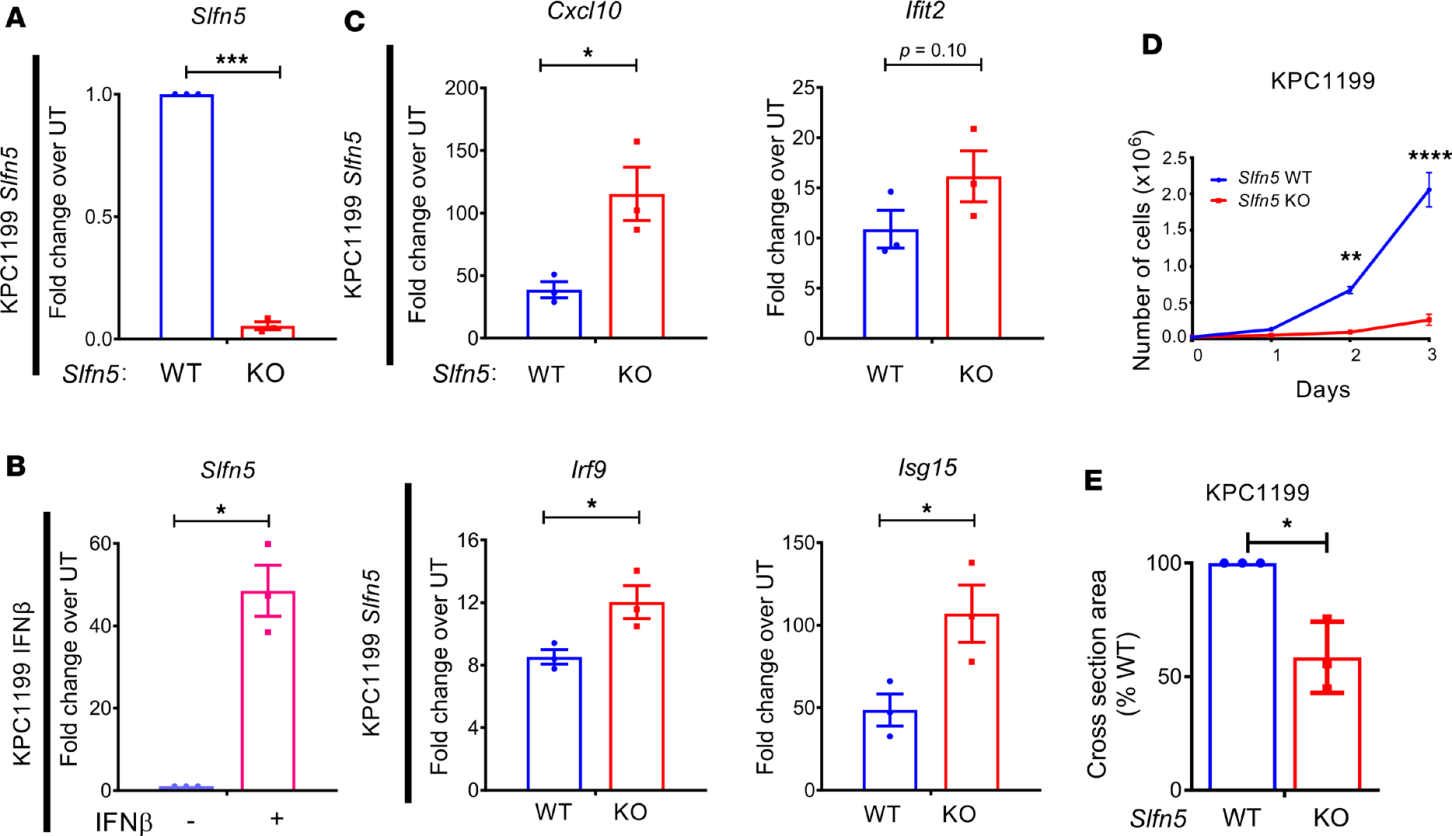
Murine *Slfn5* acts as a repressor of type I IFN signaling. (**A**–**C**) RT-qPCR analysis to monitor (**A**) efficacy of CRISPR/Cas9-mediated *Slfn5* disruption, (**B**) expression of *Slfn5* in response to IFN-β treatment (5,000 IU for 6 hours) in *Slfn5* WT cells, and (**C**) IFN-β–mediated (5,000 IU for 6 hours) induction of indicated murine ISGs in *Slfn5*-WT and *Slfn5*-KO cells. The expression levels of the indicated genes were determined using *Gapdh* for normalization and as an internal control. The data are expressed as fold change over the corresponding controls, and the graphs represent mean ± SEM of 3 independent experiments. *P < 0.05, ***P < 0.001 by 2-tailed unpaired *t* test with Welch’s correction (**A** and **B**) or 1-tailed unpaired *t* test with Mann-Whitney test (**C**). (**D**) *Slfn5*-WT and *Slfn5*-KO KPC1199 cells were plated in 6-well plates and counted on days 1, 2, and 3 after seeding. Data are mean of number of cells ± SEM of 3 independent experiments, each done in duplicate. Two-way repeated-measures ANOVA with Šidák’s multiple-comparison test; **P < 0.01, ****P < 0.0001. (**E**) *Slfn5*-WT and *Slfn5*-KO KPC1199 cells were plated into round-bottom 96-well plates under stem cell–permissive conditions to form 3D spheroids. After 7 days, spheres were imaged using a Cytation 3 cell imaging multi-mode reader to determine cross-sectional area. Data are expressed as percentages of WT parental spheres and represent mean ± SEM of 3 independent experiments, each done in triplicate. Two-tailed unpaired *t* test with Welch’s correction; *P **≤** 0.05.

Phylogenic and sequence alignment analysis suggests mouse SLFN5 represents the murine ortholog for human SLFN5 (8, 32), but to the best of our knowledge there has been no experimental evidence indicating functional overlap. As we found evidence that human SLFN5 represses ISRE-driven transcription, we sought to determine whether murine SLFN5 is similarly capable of repressing type I IFN–induced ISG transcription. We generated murine PDAC KPC1199 *Slfn5*-KO cells and confirmed efficient disruption of *Slfn5* expression (Figure 2A). Similar to human PDAC cells (3), murine *Slfn5* expression was greatly stimulated by mouse type I IFN treatment (Figure 2B). Mirroring the effects observed in human PDAC cells, mouse IFN-β–triggered expression of murine ISGs was also substantially increased after *Slfn5* loss (Figure 2C). Also similar to *SLFN5* KO in human PANC-1 and MIA-Pa-Ca-2 cells (3), loss of *Slfn5* in murine KPC1199 cells efficiently blocked cell proliferation (Figure 2D) and significantly reduced 3D spheroid growth under stem cell–permissive conditions (Figure 2E). Next, we examined whether these in vitro effects translate into anti-PDAC effects in a preclinical mouse model. Potent growth inhibitory effects were readily observed in a syngeneic flank tumor mouse model, where disruption of *Slfn5* resulted in tumor growth retardation (Supplemental Figure 2A) and significantly extended survival (Supplemental Figure 2B). Together, these results provide compelling evidence that murine SLFN5, like human SLFN5, is both IFN inducible and a modulator of type I IFN responses and promotes PDAC cell proliferation and tumor growth in vitro and in vivo.

### Loss of Slfn5 enhances type I IFN–induced antitumor effects and extends survival.

**Figure 3:**
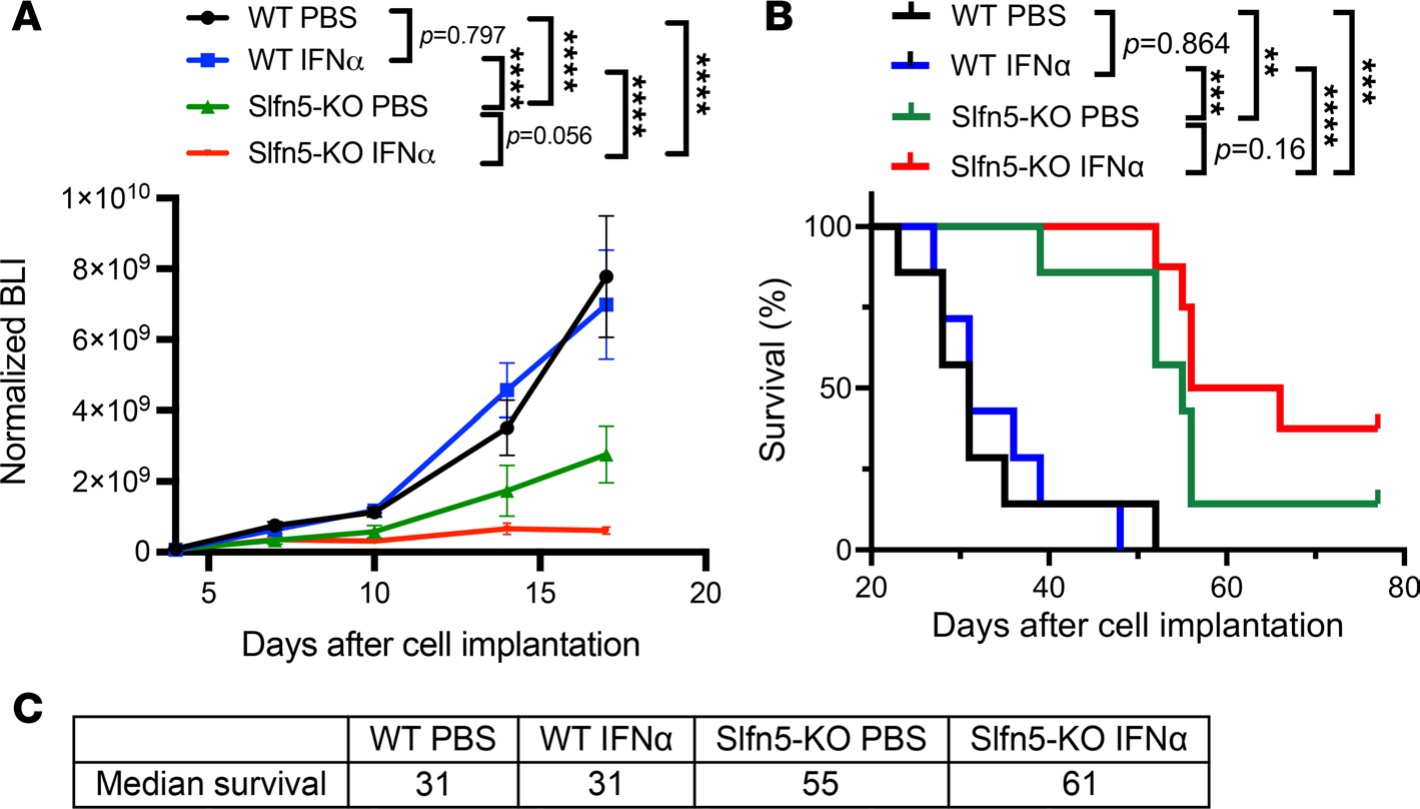
IFN-α enhances the anti-PDAC effects of *Slfn5* targeting. (**A**) KPC1199 luciferase-expressing *Slfn5*-WT or *Slfn5*-KO cells (5 × 10^4^ cells/mouse) were injected into the pancreatic tails of male and female C57BL/6J mice. Mice were randomized into the following treatment groups: *Slfn5* WT PBS (*n* = 7), *Slfn5* WT IFN-α (*n* = 7), *Slfn5* KO PBS (*n* = 7), and *Slfn5* KO IFN-α (*n* = 8). Where indicated, mice were injected subcutaneously with 600 ng murine IFN-α per mouse once per week for 2 weeks. Tumor growth was monitored at least once per week by bioluminescence imaging (BLI). Normalized BLI values are shown. On day 17, model-based estimate of the mean difference in BLI signal between WT IFN-α and *Slfn5*-KO IFN-α was 3.91 × 10^9^ photons/s/cm^2^/sr (95% CI 1.72 × 10^9^ to 6.09 × 10^9^). Two-way ANOVA with Šidák’s multiple-comparison test for day 17. Comparison of BLI signals on day 17 is based on mixed-effects model up to day 17. Data are expressed as mean ± SEM of tumor BLI signals for each genotypic treatment group; ****P < 0.0001. (**B**) Survival curves of mice bearing *Slfn5*-WT and *Slfn5*-KO pancreatic tumors. Survival was estimated using the method of Kaplan-Meier and groups were compared using the log-rank test; **P < 0.01; ***P < 0.001; ****P < 0.0001. (**C**) Median survival time (days) for indicated genotypic treatment groups was estimated using Simple Survival Analysis (Kaplan-Meier) in GraphPad.

As *Slfn5*-deficient cells exhibit enhanced transcriptional responses to type I IFNs (Figures 1 and 2), we next sought to determine whether type I IFNs can further enhance the anti-PDAC effects observed after loss of *Slfn5* (Supplemental Figure 2). While IFN-α treatment had little effect on tumor growth in mice bearing orthotopic KPC1199 *Slfn5*-WT tumors, it notably inhibited pancreatic tumor growth of *Slfn5*-KO tumors (Figure 3A). Similar effects were observed in survival rates, with IFN-α treatment extending the median survival in mice with KPC1199 *Slfn5*-KO tumors from 55 to 61 days (Figure 3, B and C). These observations indicate that loss of SLFN5 sensitizes tumors to type I IFN–mediated antineoplastic effects in vivo.

### The anti-PDAC effects of Slfn5 loss are greatly enhanced in immunocompetent hosts.

**Figure 4:**
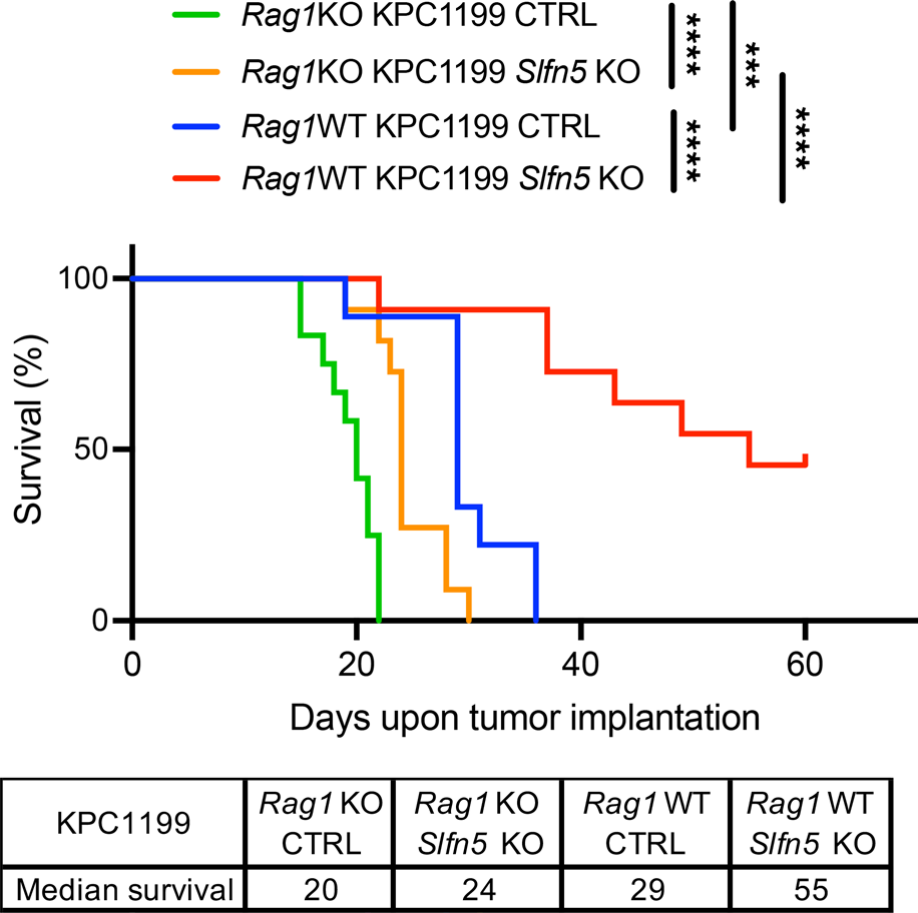
Survival benefit after *Slfn5* loss requires an intact immune system. KPC1199 luciferase-expressing CTRL or *Slfn5*-KO cells (5 × 10^4^ cells/mouse) were injected into the pancreatic tails of 6- to 8-week-old male and female mice that were grouped as *Rag1*WT + CTRL (*n* = 9), *Rag1*WT + *Slfn5* KO (*n* = 11), *Rag1*KO + CTRL (*n* = 11), and *Rag1*KO + *Slfn5* KO (*n* = 11). In the *Rag1*WT + *Slfn5* KO group, 5 mice were still alive on day 60. Survival curves of indicated mice are shown. Survival was estimated using the method of Kaplan-Meier and groups were compared using the log-rank test; ***P < 0.001; ****P < 0.0001.

As SLFN5 suppresses type I IFN transcriptional activation of immune-related genes (Figures 1 and 2), we hypothesized that it may also limit immune-mediated antitumor effects in PDAC. To determine whether immune responses account in part for the anti-PDAC effects observed after *Slfn5* loss, we employed immunocompromised *Rag1*-knockout (*Rag1*KO) mice, using their immunocompetent WT counterparts as controls. To exclude potential confounding immune-responsive effects of Cas9 expression, we generated Cas9-expressing control (CTRL) cells. We found that CTRL cells closely resembled parental WT cells in terms of cell proliferation, PDAC growth, and survival rates, and differed significantly from *Slfn5*-KO cells (Supplemental Figure 3). Orthotopic implantation of KPC1199 *Slfn5*-KO and CTRL cells into *Rag1*KO mice and their WT counterparts showed that *Slfn5* loss extended survival in both groups (Figure 4). Remarkably, immunocompetent WT mice bearing *Slfn5*-KO tumors showed the greatest survival benefit (Figure 4). These results provide strong evidence that the antitumor effects observed after *Slfn5* loss depend, at least in part, on a functional immune system.

### Remodeling of the immunosuppressive TME after loss of Slfn5 in an immunocompetent PDAC mouse model.

In PDAC, tumor cells are in constant communication with components of the TME, including immune cells (50). A hallmark of immunologically cold tumors is the greatly increased presence of suppressor cells in the TME, promoting exhaustion of effector cells and immune evasion (47, 50). Given the markedly prolonged survival observed in immunocompetent hosts (Figures 3 and 4), we next examined whether mouse SLFN5 might exert regulatory roles in the immune cell composition within the PDAC TME. To this end, we orthotopically implanted KPC1199 *Slfn5*-KO cells and CTRL counterparts in pancreases of immunocompetent C57BL/6 mice, and immune infiltrates were profiled at early (day 7) and late (day 21) time points using multicolor flow cytometric analysis.

**Figure 5:**
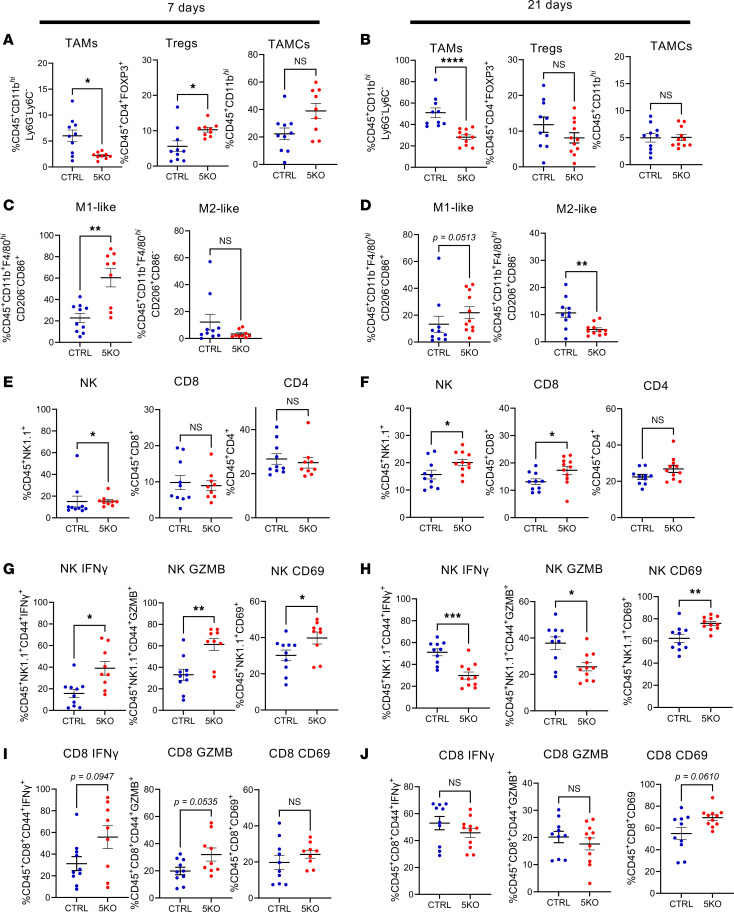
*Slfn5* loss is associated with changes within the immunosuppressive PDAC TME. (**A**–**J**) Immunophenotypic analysis of tumor-bearing pancreases by multicolor flow cytometry. (**A**, **C**, **E**, **G**, and **I**) CTRL (*n* = 10) and *Slfn5*-KO (*n* = 9) KPC1199 luciferase-expressing cells (5 × 10^4^ cells/mouse) were injected into the pancreatic tails of C57BL/6J mice and 7 days after cell implantation CTRL and *Slfn5*-KO tumor–bearing pancreases were harvested and processed for immunophenotypic analysis by multicolor flow cytometry. (**B**, **D**, **F**, **H**, and **J**) CTRL (*n* = 10) and *Slfn5*-KO (*n* = 11) KPC1199 luciferase-expressing cells (5 × 10^4^ cells/mouse) were injected into the pancreatic tails of C57BL/6J mice and 21 days after cell implantation CTRL and *Slfn5*-KO tumor–bearing pancreases were harvested and processed for immunophenotypic analysis by multicolor flow cytometry. Scatter dot plots show the percentage of tumor-infiltrating cells. (**A** and **B**) Immunosuppressive cells, i.e., TAMs (left panel), Tregs (middle panel), and TAMCs (right panel). (**C** and **D**) Innate myeloid immune cells, i.e., M1 macrophages (left panel) and M2 macrophages (right panel). (**E** and **F**) Effector cells, i.e., NK cells (left panel), CD8^+^ T cells (middle panel), and CD4^+^ T cells (right panel). (**G** and **H**) Activation markers (IFN-γ, GZMB, CD69) of NK cells, and (**I** and **J**) of CD8^+^ cells. Data are expressed as mean ± SEM of percentages of indicated immune infiltrates as detailed in Supplemental Figure 2. Two-tailed unpaired *t* test with Mann-Whitney test; *P < 0.05, **P < 0.01, ***P < 0.001, ****P < 0.0001.

Loss of *Slfn5* significantly reduced tumor-associated macrophages (TAMs) as early as day 7 (Figure 5A and Supplemental Figure 4), at a time when tumors were still comparable (Supplemental Figure 3, D and E), and this effect became more pronounced on day 21 (Figure 5B and Supplemental Figure 4). While tumor-associated myeloid cells (TAMCs) and regulatory T cells (Tregs) were transiently elevated in *Slfn5*-KO tumors at early time points (Figure 5A), both populations declined by day 21 (Figure 5B). TAMC subset analysis revealed a shift toward proinflammatory macrophages, as M1-like macrophages were significantly increased and M2-like macrophages decreased in *Slfn5*-KO tumors across both time points (Figure 5, C and D). As M1-like macrophages promote antitumor immunity whereas M2-like macrophages reinforce immunosuppression, these findings indicate that loss of *Slfn5* in cancer cells promotes a shift toward a more immunoresponsive TME.

In line with this notion, *Slfn5*-KO tumors showed increasing infiltration of effector populations over time, including natural killer (NK) and CD8^+^ T cells (Figure 5, E and F). Importantly, these cells displayed early activation in *Slfn5*-KO tumors, as evidenced by elevated expression of IFN-γ, granzyme B (GZMB), and CD69 (Figure 5, G and I). At 21 days, CD69 expression in NK and CD8^+^ T cells further increased, while GZMB and IFN-γ decreased (Figure 5, H and J). Together with extended immunophenotyping of additional immune markers (Supplemental Figure 5), these results establish SLFN5 as a driver of an immunosuppressive TME in PDAC. Disruption of *Slfn5* expression in tumor cells remodels the PDAC microenvironment toward a more immunoactive and antitumor state and this is associated with significant survival benefits (Figure 4).

## Discussion

SLFN genes are classified into 3 subgroups (I, II, and III) according to size and domain structure (32, 38, 51). While mice have 9 Slfn genes, humans have 6 SLFN genes and lack subgroup I SLFN genes. This diversity between murine and human SLFN genes raises the question about interspecies functional conservation of SLFN family members. Here, we provide compelling evidence that human SLFN5 and murine SLFN5 proteins represent functional orthologs in the context of IFN responses in PDAC. Our data demonstrate that loss of either human or murine SLFN5 greatly inhibits PDAC growth while also modulating type I IFN–dependent ISG expression. Hence, we have established syngeneic immunocompetent mouse models as suitable systems to study the roles of SLFN5. Among human SLFN proteins, the N-terminal SLFN core domain of SLFN5 is unique because it can bind dsDNA (51, 52) but lacks endoribonuclease activity (52, 53), suggesting a DNA regulatory function independent of nuclease activity. Consistent with this, SLFN5 has been implicated in the regulation of transcriptional events (3, 13) and higher-order chromatin structure (54).

In the current study, we provide strong evidence that IFN-α stimulation enhances SLFN5 enrichment on ISRE-containing ISG promoters. Furthermore, type I IFN treatment in *SLFN5*-KO cells increased endogenous ISG expression as well as exogenous ISRE-dependent luciferase expression. Mechanistically, our data indicate that type I IFNs stimulate *SLFN5* expression, eventually leading to SLFN5 association with ISRE-containing target promoter regions and resulting in transcriptional suppression of numerous ISGs. These findings strongly suggest a repressor role for SLFN5 on type I IFN downstream transcriptional responses that is conserved among human and murine SLFN5 proteins. Hence, the proposed role for SLFN5 as an intracellular immune checkpoint appears to be applicable to both human and murine SLFN5. As PDAC represents an immunologically cold tumor with limited response to current ICB therapies, extensive efforts are underway to develop strategies aimed at resensitizing PDAC to immunomodulatory approaches (55). As SLFN5 represents both a target and regulator of IFN responses in human and mouse PDAC cells, our findings strongly suggest immunocompetent syngeneic mouse models as suitable tools for studying the role of SLFN5 in anti-PDAC responses in the era of immune therapeutic approaches.

SLFN5 has been shown to have direct effects on cancer progression in different cancer types by us and others (3, 13, 15, 51, 56). However, emerging evidence suggests that its impact on cancer progression might also occur indirectly. For instance, recent findings have linked SLFN5 expression in gastric cancer with infiltration of specific immune cells such as T cells and macrophages (57). In PDAC, the immunosuppressive microenvironment is shaped and modulated by its constant crosstalk with malignant PDAC cells and additional components of the TME (58). IFNs are crucial modulators of immune responses in both malignant cells and cells of the immune system (39) and modulate immune composition of the TME (59). As SLFN5 is target and modulator of IFN responses, we sought to study the effects of *Slfn5* disruption on the immunosuppressive PDAC microenvironment. We generated immunocompetent syngeneic PDAC mouse models with *Slfn5* gene deletion. Loss of SLFN5 in KPC1199 tumors dramatically improved survival in immunocompetent WT mice, whereas only modest effects were observed in immunocompromised *Rag1*KO mice, strongly indicating that the anititumor effects are largely dependent on an intact immune system. Immune cell profiling by flow cytometry revealed that pancreases with *Slfn5*-KO tumors exhibited reduced numbers of TAMs (Ly6G^–^Ly6C^–^). Additionally, M1 macrophages were significantly elevated, while M2 macrophages decreased in *Slfn5*-KO tumors. Future mechanistic studies are warranted to elucidate potential differences in cytokine secretion, immune receptor regulation, and TME remodeling. Our observations strongly indicate that disruption of *Slfn5* can constrain components of the immunosuppressive TME, potentially allowing enhanced PDAC infiltration by effector cells. In line with this, we observed an increase in effector cells such as CD8^+^ T cells and NK cells in PDAC tumors that arose from *Slfn5*-deficient KPC1199 cells. These findings are of crucial importance because scarce infiltration of cytotoxic CD8^+^ T cells and an abundance of TAMs are characteristics of the PDAC TME, which antagonizes host anticancer immunity (47, 50). Our data demonstrate that *Slfn5* disruption promotes a favorable PDAC microenvironment, potentially triggering PDAC immune recognition by effector cells and anti-PDAC immune responses. This may prove to be of high clinical relevance, as in PDAC, the immunosuppressive TME imposes an impenetrable barrier for immune checkpoint inhibitors (ICIs) and thus is responsible for the failure of single-agent therapeutic approaches (60). Based on our findings, we propose that targeting SLFN5 in malignant cells may antagonize tumor immune evasion and facilitate ICI penetration of the immunosuppressive TME barrier. However, we found that IFN-α treatment only modestly impacted tumor growth of mice bearing orthotopic *Slfn5*-KO tumors. Future studies will be required to further determine how SLFN5 inhibition might cooperate with additional immune modulators or enhancers of type I IFN responses, such as stimulator of IFN genes (STING) agonists. Together, our findings support further exploration of the effects of combinatorial SLFN5-targeting strategies that include ICIs and IFNs. It is also possible that, as a repressor of certain type I IFN responses, SLFN5 may dampen the antitumor immune effects mediated by IFNs (59), thereby antagonizing host anticancer immunity and contributing to carcinogenesis. Consistent with this, in immunocompetent mice orthotopically implanted with *Slfn5*-KO tumors, IFN-α treatment notably reduced tumor growth and extended survival, a contrast to the less pronounced effect observed in mice with WT tumors.

In summary, we suggest that SLFN5 may be acting as an intracellular immune checkpoint and, therefore, constitutes a promising therapeutic target for further exploration in immunologically cold tumors in combination with other immunomodulatory approaches and/or IFN-based therapies. Efforts to develop SLFN5 inhibitors are currently ongoing.

## Methods

### Sex as a biological variable.

In some experiments, only female mice were used. When the studies were expanded, both male and female mice were utilized.

### Cell lines.

Human WT and *SLFN5*-KO PANC-1 and MIA-Pa-Ca-2 cells and doxycycline-inducible PANC-1-TetON-SLFN5-Myc-Flag stable cell lines were described previously (3). Murine KPC1199 cells were a gift from David Tuveson (Cold Spring Harbor Laboratory, Cold Spring Harbor, New York, USA) and propagated in DMEM supplemented with 10% FBS and antibiotics. Luciferase-expressing KPC1199 cells were generated using lentivirus with pFULT-Luciferase-Tomato as described previously (3). All cell lines were regularly tested for mycoplasma and authenticated every 6 months by STR analysis. A detailed list of cell lines and reagents can be found in Supplemental Table 3.

### Generation of mouse Slfn5-KO and CTRL cell lines.

Briefly, 2 μg of the Cas9 Control Double Nickase plasmid (Santa Cruz Biotechnology, sc437281) or 2 μg of the *Slfn5* Double Nickase plasmid (Santa Cruz Biotechnology, sc435875-NIC) were transfected into murine luciferase-expressing KPC1199 cells using Lipofectamine 2000 transfection reagent (Thermo Fisher Scientific), according to the manufacturer’s instructions. Forty-eight hours after transfection, the cells were cultured in the presence of puromycin (5 μg/mL), and 2 weeks later, the puromycin-resistant cells were expanded. Cells were seeded as single cells in 96-well plates for the generation of single clones.

### High-throughput single-end RNA-seq analysis.

Total RNA was isolated using the RNeasy Mini Kit (QIAGEN), following the manufacturer’s instructions. Library construction and stranded mRNA-seq were conducted at the NUSeq Core Facility of Northwestern University. Briefly, RNA quality and quantity were first determined with the Agilent Bioanalyzer 2100 and Qubit fluorometer, and all samples presented an RNA integrity number of 10. Sequencing libraries were prepared from 1 μg of high-quality RNA samples using a TruSeq Stranded mRNA Library Preparation Kit (Illumina), as per the manufacturer’s instructions. This procedure includes mRNA purification and fragmentation, cDNA synthesis, 3′-end adenylation, Illumina adapter ligation, library PCR amplification, and validation. An Illumina NextSeq 500 sequencer was used to sequence the libraries with the production of single-end, 75-bp reads. The quality of reads, in FASTQ format, was evaluated using FastQC v0.11.7 (https://www.bioinformatics.babraham.ac.uk/projects/fastqc/). Reads were trimmed to remove Illumina adapters from the 3′ ends using cutadapt (61). Trimmed reads were aligned to the *Homo sapiens* genome assembly GRCh38 (hg38) using STAR (62). Read counts for each gene were calculated using htseq-count (63) in conjunction with a gene annotation file for hg38 obtained from Ensembl (http://useast.ensembl.org/index.html). Normalization and differential expression were calculated using DESeq2 that employs the Wald test (64). The cutoff for determining significantly differentially expressed genes was an FDR-adjusted *P* value of less than 0.05 using the Benjamini-Hochberg method.

### Pathway enrichment analysis.

Differentially expressed gene lists were submitted to the Metascape database for gene ontology and pathway analysis, as previously described (65).

### RT-qPCR.

RT-qPCR was performed as previously described (14).

### ChIP.

ChIP using PANC-1-TetON-SLFN5-Myc-Flag cells and the SimpleChIP Enzymatic Chromatin IP Kit with Magnetic Beads (Cell Signaling Technology) was previously described (1). The following primers were used: IFIT1 FW-GGTTGCAGGTCTGCAGTTTATCTGT; IFIT1 REV-AGCTGTGGGTGTGTCCTTGC; ISG15 FW-CCACTTTTGCTTTTCCCTGTC; ISG15 REV-AGTTTCGGTTTCCCTTTCCC. RPL30 primers were included in the SimpleChIP Kit.

### Cell lysis and immunoblotting.

Cell lysis and immunoblotting were performed as described previously (14).

### Cell proliferation and 3D tumor spheroid growth assay.

For assessing cell proliferation, cells were dissociated by trypsin digestion, stained with trypan blue, and cell numbers were counted using a TC20 automated cell counter (Bio-Rad Laboratories). 3D culture and spheroid assays were described previously (3, 13).

### Flank and orthotopic tumor syngeneic models.

For subcutaneous flank implantation, cells (1.0 × 10^5^) were suspended in 100 μL of 1:1 mixture of PBS/Matrigel and then injected subcutaneously in the flank of 6- to 8-week-old C57BL/6NTac (Taconic) mice. Once tumors were palpable, tumor volume was measured at least twice per week. Tumor length and width were measured using calipers, and volume calculated according to the formula, (*D* × *d*^2^)/2, where *D* is the longest diameter and *d* is the shorter diameter. Endpoints were reached when flank tumor volume exceeded 2,000 mm^3^. For orthotopic implantation, a 25 μL cell suspension (5 × 10^4^ cells in 30% PBS and 70% Matrigel) of luciferase-expressing KPC1199 (WT, Cas9, and *Slfn5*-KO) cells was injected into the pancreatic tails of 6- to 8-week-old C57BL/6J or *Rag1*KO (B6;129S7-*Rag1^tm1Mom^*/J, The Jackson Laboratory) mice. Surgical procedure, postsurgical care, and in vivo imaging were done as described previously (3). All mice were observed until reaching IACUC endpoint criteria.

### Isolation of immune cells and cytometric immunophenotyping of mouse pancreatic tumors.

Pancreases were dissected from mice and processed as described previously (66, 67). Briefly, freshly dissected tissue was placed into a 10 cm tissue culture dish, cut into fine pieces, and incubated for 30 minutes at 37°C in a 50 mL conical tube with enzymatic digestion buffer, consisting of 4 mL of Hank’s balanced salt solution (HBSS, Gibco) supplemented with 8 mg of collagenase D (Sigma-Aldrich), 80 μg DNase I (Sigma-Aldrich), and 40 μg TLCK (Sigma-Aldrich) per approximately 2 grams of tissue. The sample was mixed by pipetting up and down several times every 10 minutes. Then, the cell suspension was mechanically dissociated using a tissue homogenizer (Potter-Elvehjem PTFE pestle) in HBSS. Cell clusters were removed using a 70 μm cell strainer (Thermo Fisher Scientific). Red blood cells, myelin, and debris were removed by 30/70 Percoll (GE Healthcare) gradient separation (30 minutes, 1200*g* at room temperature without brakes). The top layer was aspirated, and the leukocyte interphase was collected into 20 mL of ice-cold PBS and washed twice. Single-cell suspensions were incubated with Fc receptor–blocking antibody (anti-CD16/32) in FACS buffer (2% FBS in PBS) for 20 minutes at 4°C followed by staining with indicated antibodies against surface markers (see Supplemental Table 4). Next, fixable viability dye (eBioscience Fixable Viability Dye eFluor 780, Thermo Fisher Scientific) was used to exclude dead cells from subsequent analysis. For subsequent staining of intracellular antigens, cells were first fixed and permeabilized using an eBioscience Foxp3/Transcription Factor Staining Buffer Set (Thermo Fisher Scientific), according to the manufacturer’s protocol. The list of antibodies used for tumor, myeloid, and lymphocytic analysis can be found in Supplemental Table 4. All antibodies were used at a dilution of 1:100 in FACS buffer for 30 minutes at 4°C. Unstained and single-color controls were used for each experiment using fresh splenocytes isolated from naive mice and to perform compensation. OneComp eBeads Compensation Beads (Thermo Fisher Scientific) were also used for single-color compensation to establish multicolor compensation matrices. Data were acquired with a BD FACSymphony flow cytometer and analyzed using FlowJo (RRID: SCR_008520) v10.6 software.

### Definition of main immune cell subsets.

We defined specific cell subsets using the following flow cytometry phenotypes: TAMs (CD45^+^CD11b^hi^Ly6G^–^Ly6C^–^), Tregs (CD45^+^CD4^+^FOXP3^+^), TAMCs (CD45^+^CD11b^hi^), M1-like macrophages (CD45^+^CD11b^+^F4/80^hi^CD206^–^CD86^+^), M2-like macrophages (CD45^+^CD11b^+^F4/80^hi^CD206^+^CD86^–^), NK cells CD45^+^NK1.1^+^), CD8^+^ T cells (CD45^+^CD8^+^), and CD4^+^ T cells (CD45^+^CD4^+^).

### Reporter gene assay.

*SLFN5*-WT and -KO PANC-1 cells carrying the ISRE-Luciferase-pGF1 reporter vector were generated as previously described (13). Luciferase reporter assays were performed as previously described (68).

### Statistics.

Detailed statistical methodologies can be found in the figure legends. All statistical analyses were performed using GraphPad Prism 10.0 and *P* values of 0.05 or less were considered statistically significant.

### Study approval.

All animal studies were reviewed and approved by the Institutional Animal Care and Use Committee (IACUC) at Northwestern University.

### Data availability.

The data that support the findings of this study are available in the main text or the supplemental materials; values for all data points in graphs are reported in the Supporting Data Values file. RNA-seq data are available in the NCBI Gene Expression Omnibus (GEO) web portal under accession number GSE282985.

## Author contributions

Conception and design: MF, FE, MZ, and LCP. Development of methodology: MF, MZ, DS, JMM, CLC, and BAC. Acquisition of data: MF, FE, MZ, AHB, BNH, JNGM, and ELF. Analysis and interpretation of data: MF, FE, MZ, EMB, DS, GTB, PP, ETB, MK, and LCP. Writing of the manuscript: MF, FE, MZ, and LCP. Review and/or revision of the manuscript: MF, FE, MZ, and LCP.

## Funding support

This work is the result of NIH funding, in whole or in part, and is subject to the NIH Public Access Policy. Through acceptance of this federal funding, the NIH has been given a right to make the work publicly available in PubMed Central.

- NIH grant R01-NS113352.
- NIH grant R01-CA077816.

## Supplementary Material

Supplemental data

Supplemental table 1

Supplemental table 2

Supplemental table 3

Supplemental table 4

Supporting data values

## Version 1 01/27/2026

In-Press Preview

## Version 2 03/09/2026

Electronic publication
