# Supplementary material for: Schlafen 5 is an intracellular immune checkpoint and controls IFN responses in pancreatic ductal adenocarcinoma
Source: JCI Insight. 2026 Jan 27;11(5):e190031. doi: 10.1172/jci.insight.190031 (PMC13041689; doi:10.1172/jci.insight.190031)
Supplement: Supplemental data [file jciinsight-11-190031-s006.pdf]

Supplemental material for

Schlafen 5 is an intracellular immune checkpoint and controls interferon responses in pancreatic ductal adenocarcinoma

Mariafausta Fischietti[1,2,#], Markella Zannikou[1,2,#], Elspeth M Beauchamp[1,2,3], Diana Saleiro[1,2], Aneta H Baran[1,3], Briana N Hryhorysak[1], Jamie N Guillen Magaña[1], Emely Lopez Fajardo[1],Gavin T Blyth[1], Brandyn A Castro[4], Jason M Miska[1,4], Catalina Lee-Chang[1,4], Priyam Patel[1], Elizabeth T Bartom[1,5,6], Masha Kocherginsky[1,6], Frank Eckerdt[1,2], and Leonidas C Platanias[1,2,3,*.]

[1]Robert H. Lurie Comprehensive Cancer Center of Northwestern University, Chicago, IL, 60611, USA
[2]Division of Hematology/Oncology, Department of Medicine, Feinberg School of Medicine, Northwestern University, Chicago, IL, 60611, USA
[3]Department of Medicine, Jesse Brown Veterans Affairs Medical Center, Chicago, IL, 60612, USA
[4]Department of Neurological Surgery, Feinberg School of Medicine, Northwestern University, Chicago, IL, 60611, USA
[5]Department of Biochemistry and Molecular Genetics, Feinberg School of Medicine, Northwestern University, Chicago, IL, 60611, USA
[6]Division of Biostatistics, Department of Preventive Medicine, Feinberg School of Medicine, Northwestern University, Chicago, IL, 60611, USA

[#] MF and MZ contributed equally to this work.

Running Title: SLFN5, an intracellular immune checkpoint in PDAC

Financial Support: Supported by NIH grants R01-CA077816 and R01-NS113352

Keywords: Pancreatic ductal adenocarcinoma (PDAC), Schlafen 5 (SLFN5), Interferon (IFN)

***Corresponding author:** Leonidas C. Platanias**;** 303 East Superior Street, Lurie- 3125, Chicago, IL 60611; email: l-platanias@northwestern.edu

Conflict of Interest Statement: No potential conflicts of interest were disclosed related to the content of this manuscript.

Supplementary Figure S1

A

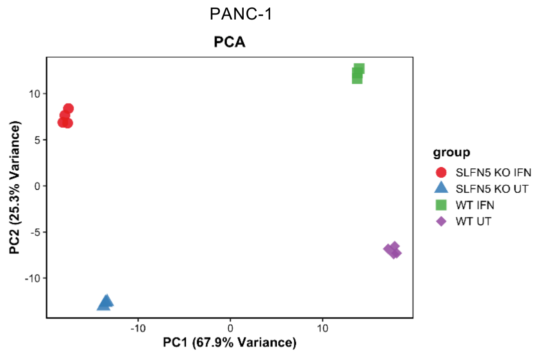

B

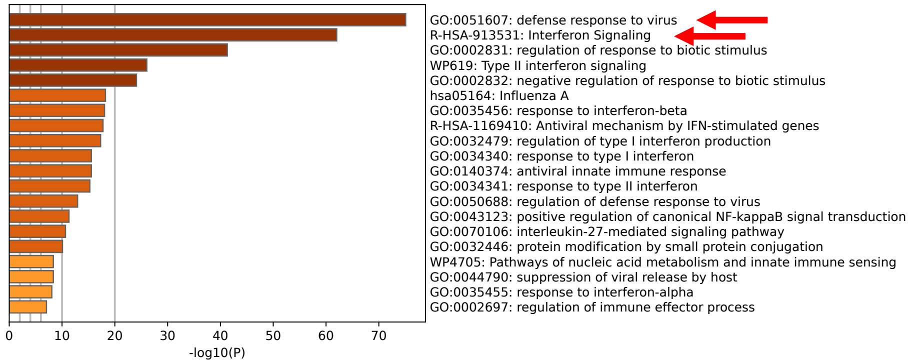

<u>Figure S1.</u> IFNα induces transcription of ISGs in pancreatic cancer cells. (A,B) RNA-seq analysis of transcript expression in *SLFN5* WT and *SLFN5* KO PANC-1 cells untreated or treated with human IFNα (5000 IU) for 6 hours. **(A)** Principal Component Analysis (PCA) of indicated groups. **(B)** Ontology analysis of the 199 genes that were found to be ≥ 2-fold higher expressed in IFNα treated *SLFN5* KO cells. Note that red arrows highlight “defense response to virus” and “Interferon Signaling” pathways.

Supplementary Figure S2

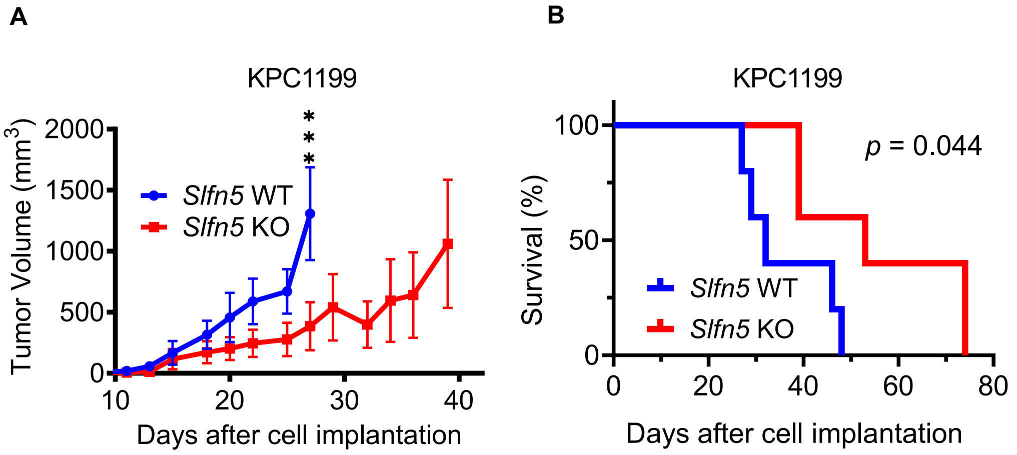

Figure S2. Genetic deletion of *Slfn5* results in potent anti-tumor effects. (A) *Slfn5* WT (n=5) and *Slfn5* KO (n=5) KPC1199 cells (1 x 10^5 cells/mouse) were injected subcutaneously into the right flank of 6-8 weeks old C57BL/6NTac female mice (Taconic). *Slfn5* WT and KO tumor volumes are shown until the day the first mouse of each group had to be euthanized. On day 27, model-based estimate of the mean difference in tumor volume between groups was 921.2 mm^3 (95% CI: 342 to 1500). Two-way ANOVA with Sidak's multiple comparison test for day 27. Comparison of tumor volumes on day 27 is based on Mixed-effects model up to day 27. Data are expressed as means ± SEM of tumor volumes for each genotypic group; ***, $p = 0.0002$. **(B)** Kaplan-Meier survival curves of mice as in (A). Survival was estimated using the method of Kaplan-Meier and groups were compared using the log-rank test; *, $p = 0.0446$.

Supplementary Figure S3

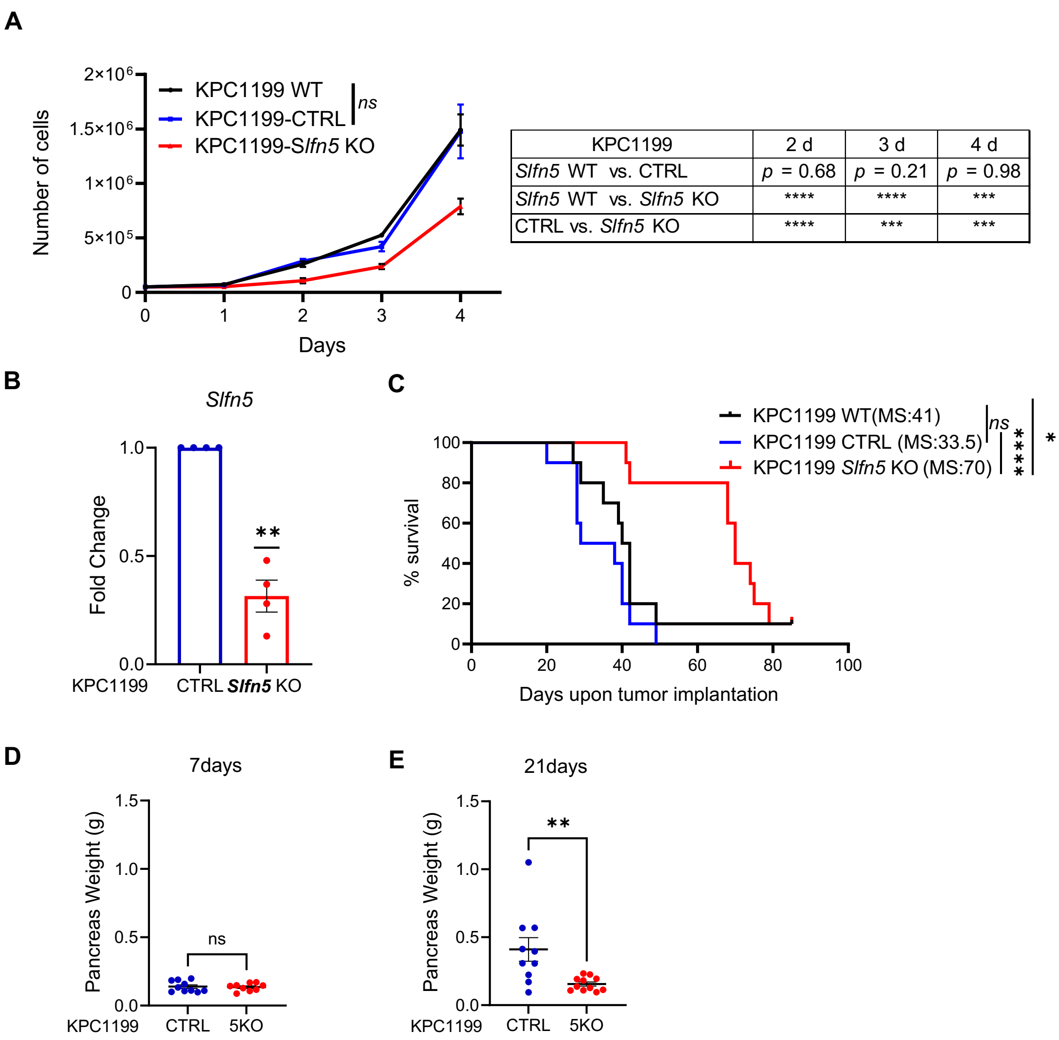

KPC1199	2 d	3 d	4 d
Slfn5 WT vs. CTRL	p = 0.68	p = 0.21	p = 0.98
Slfn5 WT vs. *Slfn5* KO	****	****	***
CTRL vs. *Slfn5* KO	****	***	***

Figure S3. Generation and characterization of Cas9 CTRL KPC1199 cells. **(A)** Luciferase-expressing KPC1199 WT, CTRL or *Slfn5* KO cells were plated in 6-well plates and counted at days 1, 2, 3 and 4 after seeding. Data are means of number of cells ± SEM of three independent experiments, each done in duplicate. Statistical analysis was performed using ordinary two-way analysis of variance (ANOVA) with time and treatment group as predictors, followed by Tukey's multiple comparisons test and comparisons are shown in the table. The outcomes were log2-transformed to satisfy the normality assumption; ***, $p < 0.001$; ****, $p < 0.0001$. **(B)** Quantitative PCR (RT-qPCR) analysis to monitor efficacy of CRISPR/Cas9 mediated *Slfn5* disruption. Data are expressed as means ± SEM of four independent experiments and are represented as fold change normalized to their corresponding Cas9 control (CTRL). Statistical analysis was contacted using a One–sample student's *t*-test compared to a fold change of 1; **, p = 0.0027. **(C)** Luciferase-expressing KPC1199 WT (n=10), CTRL (n=10), or *Slfn5* KO (n=10) cells (5 x 10^4 cells/mouse) were

injected into the pancreatic tails of 6-8 weeks old male and female C57BL/6J mice. Survival curves of indicated mice are shown. Survival was estimated using the method of Kaplan-Meier and groups were compared using the log-rank test; *, p= 0.0497 ; ****, $p < 0.0001$. **(D,E)** Mouse pancreatic weight (g). KPC1199 CTRL or *Slfn5* KO cells (5×10^4 cells/mouse) were injected into the pancreatic tails of 6-8 weeks old C57BL/6J mice (males and females) and after either 7 days (CTRL, n=10; *Slfn5* KO, n=9) or 21 days (CTRL, n=10; *Slfn5* KO, n=11) the pancreatic weight was assessed. One-tailed unpaired *t*-test with Mann-Whitney test; **, $p < 0.01$.

Supplementary Figure S4

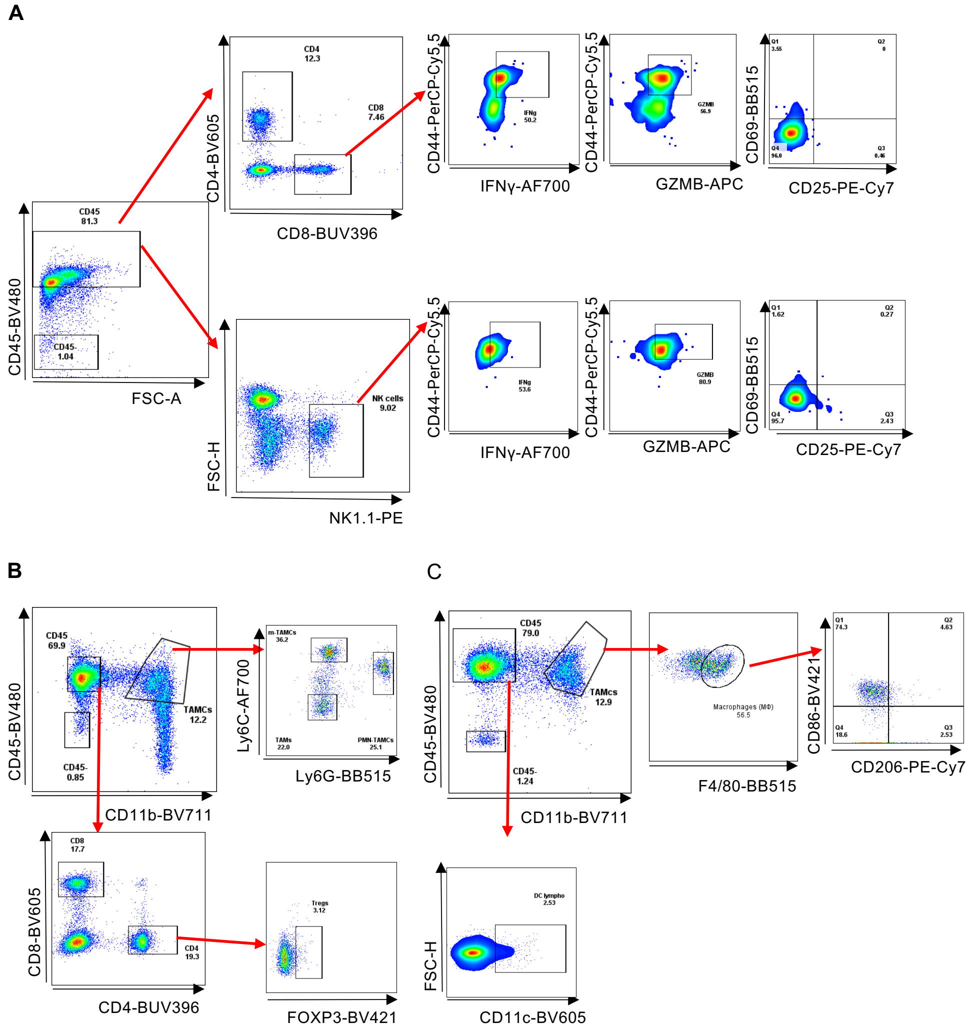

<u>Figure S4.</u> Gating strategy for flow cytometric immunophenotyping of mouse pancreatic tumors. (A) Representative images of the flow cytometric gating strategy used to assess the percentages of $CD45^+$ cells, CD4, CD8, NK cells in KPC1199 pancreatic tumors shown in figure 4A using anti-CD45-BV510, CD8-BV605, CD4-BUV396, and NK1.1-PE antibodies with further analysis of IFNγ-, granzyme B-, CD69-, and CD25- expression using CD44-PerCP-y5.5, IFNγ-AF700, GZMB-APC, CD69-BB515 and CD25-PE-Cy7. **(B)** Representative images of the flow cytometric gating strategy used to assess the percentages of CD45+ cells, TAMCs, m-TAMCs, PMN-TAMCs and TAMs using anti- CD45-BV510, CD11b-BV711, Ly6G-BB515 and Ly6C-AF700 antibodies and the percentages of Tregs, CD8+ T cells, and CD4+ T cells using CD8-BV605, CD4-BUV396, and FOXP3-BV421. **(C)** Representative images of the flow cytometric gating strategy used to assess the percentages of CD45+ cells, TAMCs, Macrophages(MΦ),M1 MΦ, M2 MΦ and dendritic cells (DC lympho) in KPC1199 pancreatic tumors using anti-CD45-BV480, CD11b-BV711, macrophages-F4/80-BB515, M1 MΦ-CD86-BV421, M2 MΦ-PE-Cy7 and DC lympho-BV-605.

Supplementary Figure S5

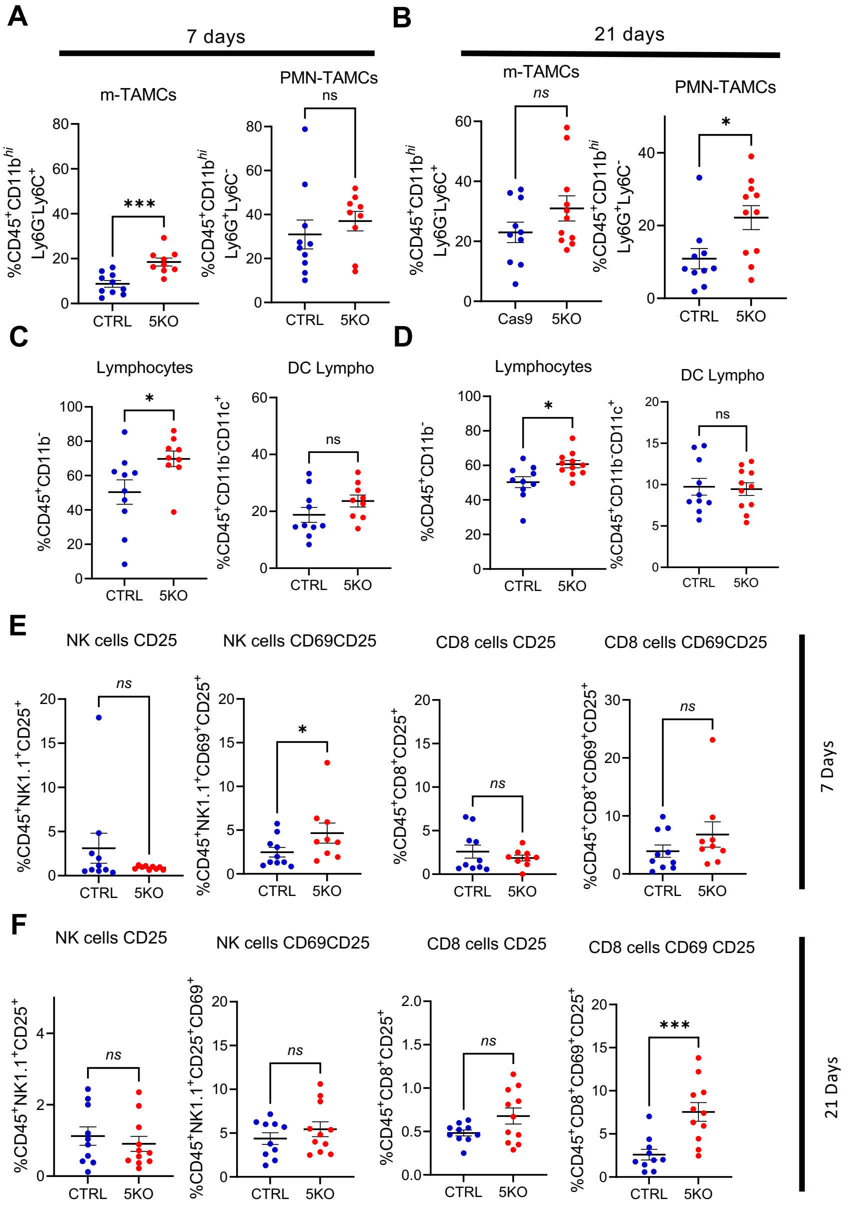

<u>Figure S5.</u> Loss of *Slfn5* is associated with alterations within the immune-suppressive PDAC TME. (A-F) Immunophenotypic analysis of tumor-bearing pancreases by multicolor flow cytometry. **(A, C, E)** *Slfn5* CTRL (n=10) and *Slfn5* KO (n=9) luciferase-expressing KPC1199 cells (5 x 10^4 cells/mouse) were injected into the pancreatic tails of C57BL/6J mice, and 7 days after cell implantation CTRL and *Slfn5* KO tumor-bearing pancreases were harvested and processed for immunophenotypic analysis by multicolor flow cytometry. **(B, D, F)** CTRL (n=10) and *Slfn5* KO (n=11) luciferase-expressing KPC1199 cells (5 x 10^4 cells/mouse) were injected into the pancreatic tails of C57BL/6J mice and 21 days after cell implantation, CTRL and *Slfn5* KO tumor bearing pancreases were harvested and processed for immunophenotypic analysis by multicolor flow cytometry. Scatter dot plots show the percentage of tumor-infiltrating cells. **(A,B)** immunosuppressive cells, i.e. m-TAMCs (left panel) and PMN-TAMCs (right panel). **(C, D)** i.e., lymphoid cells (left panel) and dendritic cells (right panel). **(E)** activation markers (CD69, CD25) of NK and CD8 T cells 7 days upon implantation. **(F)** activation markers (CD69, CD25) of NK and CD8 T cells 21 days upon implantation. Data are expressed as means ± SEM of percentages of indicated immune infiltrates as detailed in supplementary figure S2. Two-tailed unpaired *t*-test with Mann-Whitney test ; *, $p < 0.05$; **, $p < 0.01$; ***, $p < 0.001$; ****, $p < 0.0001$.
